# The stochastic frontier analysis technique in measuring the technical and economic efficiency of hospital diagnostic laboratories: a case study in Iran

**DOI:** 10.1186/s12962-022-00406-8

**Published:** 2022-12-07

**Authors:** Siamak Aghlmand, Sadegh Feizollahzadeh, Behrouz Fathi, Hasan Yusefzadeh, Mina Alinejhad

**Affiliations:** 1 Department of Health Economics and Management, School of Public Health, Urmia University of Medical Sciences, Urmia, Iran; 2 Department of Medical Laboratory Sciences, School of Allied Medical Sciences, Solid Tumor Research Center, Cellular and Molecular Medicine research Institute, Urmia University of Medical Sciences, Urmia, Iran

**Keywords:** Hospital, Diagnostic laboratory, Stochastic frontier analysis, Economic efficiency

## Abstract

An inefficient health system wastes scarce resources even if it makes considerable gains in accountability and equity. Such a system is expected to perform better. Therefore, it is vital to examine the current performance of health systems and their constituents and assess how to reach their maximum potential. This study aimed to evaluate the technical and economic efficiency of medical diagnostic laboratories in hospitals affiliated with Urmia University of Medical Sciences (UUMS) in 2016. In this descriptive-analytical study, data from diagnostic laboratories of the hospitals of UUMS have been inputted into Frontier_4.1_ software after taking the log of variables. Then, the technical and economic efficiency of the laboratories were obtained by estimating the production and cost function using the stochastic frontier analysis method, assuming input minimization for 2016. The mean technical and economic efficiency score of the diagnostic laboratories were determined to be 93.1% and 51.9%, respectively. These laboratories need to reduce their inputs and costs in order to achieve full efficiency without changing the amount of their output. Although the average economic efficiency of the diagnostic laboratories of the studied hospitals was high, there is still an increase in the efficiency of these units, given the cost of inputs at the time of allocating resources. In addition, it is possible to improve the technical efficiency of the clinical laboratories of hospitals affiliated with UUMS by 48.1% by applying the same level of inputs and without increasing the costs.

## Introduction

Measuring the performance of hospitals’ clinical laboratories as an important and costly unit is essential for their managers [1] as they can demonstrate its strengths and weaknesses, identify areas that need improvement, and increase productivity in the hospital [2].

A laboratory is where various operations such as empirical tests, measurements, and the analysis and identification of materials and impurities are performed [3]. In this regard, medical diagnostic laboratories play an essential role in providing high-quality care to patients [4]. Now that communities have recognized the value of health, it is impossible to maintain community health, prevent the spread of infectious diseases, and combat genetic diseases without medical diagnostic laboratories [5].

The laboratory is a sensitive and vital work environment, employing specialized laborers in various fields and costly equipment. Its performance and efficiency require a great deal of attention [6]. In this regard, economic efficiency demonstrates the ability of the laboratory to obtain maximum benefit according to price and input levels [7]. Economic efficiency is associated with a combination of technical efficiency and allocative efficiency [8]. Technical efficiency indicates the ability of the laboratory to maximize the product (service delivery) concerning specific production factors or to minimize production factors regarding the particular product [9]. Allocation efficiency is also the allocation of limited resources to different inputs to maximize output [10].

A laboratory has economic efficiency only if it maximizes technical and allocation inputs. Economic efficiency can be achieved if the best use is made of resources in the laboratory unit without wasting the resources [11]. It requires inputs with the lowest cost. Economic efficiency is obtained by multiplying the quantities of technical and allocative efficiencies.

Different methods are used to evaluate the performance of service and manufacturing units. These methods are generally divided into nonparametric and parametric categories [12]. One of the most common parametric methods is stochastic frontier analysis (SFA), in which statistical disturbances are considered. It is necessary to consider assumptions about the function’s frontier form. Statistical disturbances or noise terms are stochastic variables and factors beyond the firm’s control, such as weather, device failure, measurement error, and strikes. This method is an econometric technique that identifies deviation from the best performance frontier and demonstrates the effect of the noise term on efficiency, which is beyond the control of production units. This feature divides the deviation of the frontier into two components: inefficiency and random error [13]. In this method, the production function is estimated as the maximum product that can be produced from a set of production factors and provides a better definition of inefficiency based on economic theory. The SFA is appropriate if the rate of production factors and production is a random mechanism [14, 15].

The studies of Alinejad et al. [1], Taheri [16], and Lamovsek [4] have investigated the performance and efficiency of laboratories through methods of Data Envelopment Analysis and Stochastic Frontier Analysis.

## Objectives

Studying the performance of clinical laboratories and identifying their strengths and weaknesses are of great importance in the optimal allocation of facilities. The present study evaluates the efficiency of resource use in clinical laboratories of hospitals affiliated with Urmia University of Medical Sciences (UUMS) in 2017 via the SFA method. The results can help hospital managers improve laboratory units’ economic performance by avoiding the waste of scarce resources and thus reducing unit costs.

## Methods

This descriptive-analytical research was conducted in Urmia city, northwest Iran, in 2017. In the present study, the technical and economic efficiency of 22 diagnostic laboratories of the hospitals affiliated with the UUMS were estimated using the SFA method by the variable returns to scale (VRS) and input-oriented assumptions via Frontier_4.1_ software. Return to scale shows the rate of increase in production provided that all other resources are equally increased. When the production mix is a combination of increasing, decreasing, or constant returns, the returns to scale will be variable.

The general form of the Cobb–Douglas production function to calculate the technical efficiency of the laboratory units in this study is as follows [17]:

$$\mathrm{Ln }{Y}_{it}= {\beta }_{0}+\sum {\beta }_{j}{X}_{jit}+({V}_{it}-{U}_{it})$$

$$Ln\left({Y}_{it}\right)={\beta }_{0}{+\beta }_{1}Ln\left({P}_{it}\right)+{\beta }_{2}Ln\left({E}_{it}\right) +{\beta }_{3}Ln\left({T}_{it}\right){+\beta }_{4}Ln\left({I}_{it}\right)+{\beta }_{5}Ln\left({S}_{it}\right)+({V}_{it}-{U}_{it})$$

where Ln: logarithm at the base of natural number, Y_it_: production of unit i at time t, X_jit_: rate of using production factor j by unit i at time t, V_it_: random disturbance component, and U_it_: model inefficiency. Inputs include the number of specialists (P), experts (E), technicians (T), tools and equipment (I), and materials and solutions (S), and the output contains the number of patients admitted to the laboratory unit (Y).

The general form of the Cobb–Douglas cost function for estimating the economic efficiency of the laboratory units by the SFA and Maximum Likelihood Method (MLE) is as follows [17]. MLE is a method to estimate the parameters of a statistical model. The MLE method consists of assigning values to the parameters of the model, resulting in a distribution that gives the highest probability to the observed data (that is, parameter values that maximize the likelihood function).

$$\mathrm{Ln }\left({C}_{it}/{W}_{Pit}\right)= {\beta }_{0}+{\beta }_{1}Ln\left({Y}_{it}\right)+{\beta }_{2}Ln\left(\frac{{W}_{Eit}}{{W}_{Pit}}\right){+\beta }_{3}Ln\left(\frac{{W}_{Tit}}{{W}_{Pit}}\right)+{\beta }_{4}Ln\left(\frac{{W}_{Iit}}{{W}_{Pit}}\right) +{\beta }_{5}Ln\left(\frac{{W}_{Sit}}{{W}_{Pit}}\right)+({V}_{it}-{U}_{it})$$

where Ln: logarithm at the base of natural number, C_it_: total cost, Y_it_: number of patients admitted, W_Pit_: specialist wages, W_Eit_: expert wages, W_Tit_: technician wages, W_Iit_: the price of tools and equipment, W_Sit_: the price of materials and solutions, V_it_: random disturbance component, and U_it_: model inefficiency. To calculate the total cost, the costs of annual properties (including medical and non-medical), construction, consumables, equipment, and salaries of all employees in the laboratory unit, which are a valid representation of the total cost of that unit, were used. Moreover, part of the costs of the laboratory unit is the overhead costs that are split or shared between different parts of the hospital.

The price of the equipment is its annual depreciation expense, and the straight-line method was used to calculate the depreciation of equipment.

Also, in the SFA method, the obtained numbers for economic efficiency were divided into the highest economic efficiency number so that the most efficient laboratory unit got number 1, and the other units will be below one. This allowed for the easy comparison of efficient and inefficient units’ technical and economic efficiency [18].

## Results

Table 1 summarizes the results of the stochastic frontier cost function estimation. Since the index of the likelihood ratio (LR) test was high in this estimate for the Cobb–Douglas function, there was no need to estimate the translog function. In order to calculate the economic efficiency, it was necessary to estimate the cost function through the cost of inputs and the total cost of the laboratory unit. In this study, the total costs of construction, consumables, property, equipment, and staff salaries of the relevant laboratory were used to calculate the total cost of the hospitals’ laboratory units.

**Table 1:** Estimation of Frontier Cost Function Parameters (SFA) from the maximum likelihood method (ML)

Variable	Coefficient		Standard-error	t-ratio
Intercept	β_0_	3.86	0.925	4.175
log(expert)	β_1_	0.22	0.819	0.275
log(specialist)	β_2_	3.82	0.478	7.986
log(auto‐analyzer)	β_3_	− 1.54	0.585	− 2.63
log(ELISA)	β_4_	− 0.7	0.495	− 1.42
log(cell counter)	β_5_	0.58	0.567	1.028
log(incubator)	β_6_	0.15	0.39	0.381
log(centrifuge)	β_7_	− 0.62	0.657	− 0.95
log(microscope)	β_8_	0.55	0.794	0.692
log(lubricating solution)	β_9_	− 1.02	0.492	− 2.08
log(isotone solution)	β_10_	1.31	0.575	2.282
log(Hormone kit)	β_11_	− 1.45	0.503	− 2.88
log(Biochemistry Kit)	β_12_	1.27	0.448	2.828
log(microbial culture medium)	β_13_	− 0.63	0.345	− 1.82
Sigma-square	σ^2^	1.14	0.214	5.333
Gamma	γ	1	0.0000006	1,660,708.2
log likelihood	− 20			
LR test	7.93			

In Column 2 of Table 2, the economic efficiency obtained from the cost function estimation is greater than one because Frontier_4.1_ does not consider a constraint in the cost function estimation, such as the economic efficiency range between zero and one. In order to compare the calculated SFA efficiencies, the economic efficiency figures obtained from the SFA method were divided into the highest cost efficiency figure in Table 2 (1.124) until the numerical values of economic efficiency fall between zero and one (Column 3 in Table 1). In this method, the average economic efficiency of the laboratory units of hospitals affiliated with the UUMS was 0.931 (SD = 0.034). Also, the lowest cost-efficiency belonged to Hospital Laboratory 9 (value = 0.89), and the highest cost efficiency was related to Hospital 13 (value = 1).

**Table 2:** Economic efficiency of clinical laboratories of hospitals affiliated with the UUMS through the SFA method

Laboratory of hospital	Economic efficiency (2)	Economic efficiency (3)
1	1.013	0.901
2	1.119	0.996
3	1.015	0.903
4	1.024	0.911
5	1.111	0.988
6	1.103	0.981
7	1.009	0.898
8	1.037	0.923
9	1	0.89
10	1.044	0.929
11	1.028	0.914
12	1.065	0.948
13	1.124	1
14	1.032	0.918
15	1.045	0.93
16	1.066	0.949
17	1.046	0.93
18	1.068	0.95
19	1.016	0.904
20	1.003	0.892
21	1.039	0.925
22	1.02	0.908
Mean	1.047	0.931

In Table 3, no significant relationship is observed between output, i.e., the number of admissions, with expert, ELISA, cell counters, incubators, centrifuges, microscopes, and microbial culture medium inputs. In other words, these inputs do not considerably impact the output level, which may be due to the high similarity of data collected from hospitals’ laboratories and, therefore, a reduced fluctuation between data and their low variance. Also, in Table 3, the sum of the partial elasticity coefficients of inputs was 1.94, so the laboratory units of the studied hospitals had an increasing return to scale. The negative production elasticity of some production factors indicated that the laboratories under study were in the third and non-economic stage of production. At this stage, the increase in labor and capital will lead to a decrease in production. Also, the production elasticity relative to specialist input was 3.82, which was greater than the other elasticities. This means that a one percent increase in this production factor leads to the highest increase in the return of laboratories by 3.82%. According to the final results of the maximum likelihood estimation regarding the accuracy of using the Cobb–Douglas function, since the LR value was 7.93, the Cobb–Douglas function form was chosen. In other words, this form was suitable for the SFA for the studied laboratory units due to the high LR value of the Cobb-Douglas function rather than the translog function. The gamma variable that shows the contribution of inefficiency variance in the production function also equaled 1 with a standard error of 0.0000006. That is, the share of stochastic factors in the inefficiency of clinical laboratories in hospitals affiliated with the UUMS was equal to zero, and the variables included in the studied model impacted the inefficiency rate most. The significance of the γ parameter confirms that inefficiency plays an important role in the model.

**Table 3:** Estimation of stochastic frontier production function (SFA) parameters from the maximum likelihood method (ML)

Variable	Coefficient		Standard-error	t-ratio
Intercept	β_0_	1.27	0.039	32.6
log(number of admissions)	β_1_	0.014	0.005	2.72
log(tools and equipment cost/ salaries of all experts)	β_2_	0.31	0.017	18.4
log(tools and equipment cost/ salaries of all specialists)	β_3_	0.33	0.016	21.17
log(cost of materials and solutions/ salaries of all specialists)	β_4_	0.092	0.012	7.62
Sigma-square	σ^2^	0.003	0.0007	4.2
Gamma	γ	0.99	0.000004	266,875
log likelihood	47.02			
LR test	7.03			

According to Table 4, the average technical efficiency of medical diagnostic laboratories affiliated with the UUMS was 0.519 (SD = 0.33). According to the SFA model, the lowest technical efficiency belonged to Hospital 17 (value = 0.049), and the highest technical efficiency belonged to Hospitals 2 and 3 (value = 0.999).

**Table 4:** Technical efficiency of the clinical laboratories of hospitals affiliated with the UUMS through the SFA method

Laboratory of hospital	Technical efficiency
1	0.998
2	0.999
3	0.999
4	0.251
5	0.33
6	0.598
7	0.182
8	0.376
9	0.103
10	0.584
11	0.608
12	0.246
13	0.333
14	0.998
15	0.422
16	0.419
17	0.049
18	0.432
19	0.334
20	0.959
21	0.999
22	0.211
Mean	0.519

## Discussion

The purpose of efficiency measurement is to provide the best possible service in a practical, timely, humane, and economically efficient way. Efficiency is the result of optimizing production costs and optimal allocation of resources. Based on the estimated technical and economic efficiency scores, suggestions can be made to maximize efficiency by changing the number and combination of inputs and outputs.

The technical and economic efficiency of medical diagnostic laboratories of hospitals affiliated with the UUMS was estimated via SFA under VRS and input-oriented minimization assumption by estimating production and cost functions to evaluate their performance.

The low technical efficiency of the medical diagnostic laboratories of the hospitals indicated the high capacity of inputs in these units. While using the same level of inputs, these units can increase their outputs and thus their technical efficiency by 48.1% without any cost increase.

The average economic efficiency of the units mentioned above was 0.931. Therefore, there is a capacity to improve the efficiency of these units by reducing costs by 6.9% without decreasing the output level. In other words, the managers of these laboratories could reduce their costs by optimally combining and distributing inputs while considering the prices of inputs to approach the profitability frontier.

The average economic efficiency of laboratory units was higher than the average technical efficiency, demonstrating the high allocative efficiency of these units; that is, the combination of inputs in these units is appropriate. However, service providers in these units can make the most of this combination to increase service delivery without changing the amount of inputs to increase technical efficiency. Therefore, to reduce costs and increase their profits, the laboratory mentioned above units should avoid wasting resources by enhancing the motivation of the staff and the awareness of the general principles of medical diagnostic laboratories.

Furthermore, the medical diagnostic laboratories of university hospitals had an increasing return to scale. In other words, the coefficient of function or degree of homogeneity was > 1; if inputs are increased by 1%, the output of laboratories will increase by more than 1%. Therefore, the laboratories under study should increase their supply of services.

In the study of Alinejad et al., the average economic efficiency of clinical laboratories in Iran’s public hospitals via the DEA method was 67.6% [1]. According to the findings of two efficiency measurement methods, i.e., DEA and SFA, the economic efficiency values calculated for the hospital’s laboratory units of the hospitals affiliated with UUMS were different. This difference can be caused by not considering the influence of stochastic factors and measurement errors in the DEA method. In general, however, the average economic efficiency of the studied units in the province is medium to high, indicating that in the studied units, it is possible to increase economic efficiency by reducing costs without affecting output values. In the study of Lamovsek et al., the average technical efficiency of the laboratories was equal to 93.33%, and the increase in automation and consolidation of laboratory activities can affect the efficiency of the laboratories and thus the costs [4].

In evaluating the technical efficiency of the clinical laboratories of Shiraz University of Medical Sciences, Taheri et al. reported the average technical efficiency of laboratory units to be 0.924 [16], which indicates a high level of technical efficiency in hospital units of Shiraz compared to Urmia. It means that Shiraz’s laboratory units used their resources more optimally.

In general, SFA is a valuable tool for analyzing the efficiency of medical diagnostic laboratories, and its information may be helpful for the policy-making and performance evaluation of these units. The DEA and SFA methods are also complementary in efficiency measurement [13, 19, 20]. However, for efficiency measurement, it is recommended that the SFA method be first performed to identify the variables having an adverse effect and exclude them from the model.

One of the limitations of the present study was the lack of information on private-sector diagnostic laboratories and their number in the country. Better analysis and conclusions could be obtained from the efficiency status of provincial laboratories had these laboratories cooperated, and the health planners in the province could have used better data for decision making.

The results above could be applied in hospitals as a benchmark for decision-making about resource allocation, monitoring, and improving public hospital performance.

## Conclusions

The results of this research show that the performance level of the laboratories is far from the ideal level, so these units should use their inputs more efficiently to get closer to the desired performance level. By reducing the extra inputs based on the results obtained from the SFA and DEA methods, it is possible to help decrease the costs of the healthcare sector in Iran. Moreover, the results of these methods can be used as a criterion for the optimal allocation of resources, control, and improvement of the health system’s performance.

Using the SFA method to analyze the technical and economic efficiency of clinical diagnostic laboratories in Iran and other countries can help improve laboratories' efficiency and reduce their costs by suggesting how to use resources and manage them better. In addition, efficiency measurement allows inefficient laboratories to compare their efficiency scores and resources with similar but efficient laboratories and identify their efficiency promotion capabilities by pursuing efficient laboratories and using their resources optimally.

## Data Availability

The essential data is available in the article and we can provide upon request.

## Abbreviations

SFA: Stochastic frontier analysis
DEA: Data envelopment analysis
